# Effective Contact Tracing for COVID-19 Using Mobile Phones: An Ethical Analysis of the Mandatory Use of the Aarogya Setu Application in India

**DOI:** 10.1017/S0963180120000821

**Published:** 2020-09-30

**Authors:** SAURAV BASU

**Keywords:** contact tracing, contact tracing applications, Aarogya Setu, India, public health ethics, COVID-19, pandemic ethics

## Abstract

Several digital contact tracing smartphone applications have been developed worldwide in the effort to combat COVID-19 that warn users of potential exposure to infectious patients and generate big data that helps in early identification of hotspots, complementing the manual tracing operations. In most democracies, concerns over a breach in data privacy have resulted in severe opposition toward their mandatory adoption. This paper examines India as a noticeable exception, where the compulsory installation of such a government-backed application, the “Aarogya Setu” has been deemed mandatory in certain situations. We argue that the mandatory app requirement constitutes a legitimate public health intervention during a public health emergency.

## Background

To date, no effective vaccine and no licensed medication for protection or cure is yet available against the global pandemic caused by the Coronavirus 2 (COVID-19), a disease with high transmission potential that spreads through respiratory droplet infection.^1^ Contact tracing constitutes a key mechanism for breaking the chain of disease transmission by identifying, locating, and assessing contact-people exposed to a COVID-19 positive case. Trained healthcare workers interview the confirmed or suspected patients to identify all their contacts during the likely period of communicability, and quarantining, testing, and isolating them in a continuous loop. Furthermore, effective contact-tracing allows the early detection of hotspots and clusters, and their sanitization and geographical containment.^2^

Nevertheless, it is well-established that contact tracing for infectious diseases during pandemics involves several challenges.^3^ ^,^^4^ Mistrust of contact tracing personnel due to perceived stigmatization in the community may sometimes render it difficult to identify the contact-persons, some of whom may refuse to disclose their identity and addresses. Logistic challenges in identifying the contact-persons in areas like dense-neighborhoods, busy markets, and travel through mass public transport are evident. Contact tracing personnel can also become demotivated and inefficient if they perceive themselves to be at higher risk of contracting the disease, especially if not provided with appropriate personal protective equipment. Furthermore, during the COVID-19 pandemic, instances of health staff facing violent attacks during community surveillance activities have been occasionally reported.^5^ Finally, contact tracing in COVID-19 is complicated by the shedding of the virus even before the development of symptoms, and by some who are chronic asymptomatic carriers.^6^

There is growing recognition that digital technology can complement contact tracing operations for infectious diseases.^7^ ^,^^8^ Moreover, these applications also enable a system of participatory disease surveillance wherein the users are expected to self-report specific symptoms to assist public health personnel in analyzing and responding to the emerging health threats.^9^ The feasibility of implementing such technology is also driven by the exponential worldwide growth of mobile phone and information technology.

Several automated contact tracing applications have been developed by countries globally in their efforts to combat COVID-19, which can notify the user of any potential exposure to a COVID-19 infected patient.^10^ Furthermore, the big data generated on movement patterns and syndromic mapping can catalyze the efforts of public health officials in identifying and predicting the growth of hotspots or clusters of patients through epidemiological modeling and implement effective interventions for preventing or containing the spread of the disease. Digital contact tracing also has the advantage of foolproof recording of the user’s movements since it is possible that some people who test COVID-19 positive may not recall all their known and unknown contacts, especially when travelling through public transport, visiting busy markets, or when participating in large gatherings.

However, these applications differ with regard to their underlying technologies, their modes, and the extent of data collection.^11^ Although the degree of efficacy of these contact tracing applications in improving the tracking of COVID-19 cases remains unclear, there is emerging evidence of their utility in identifying hotspots of the disease and sending real-time alerts to users having potential exposure to the severe acute respiratory syndrome coronavirus 2 (SARS-CoV-2).^12^

Nevertheless, despite these advantages, digital proximity or contact tracing applications have been viewed with suspicion by civil liberty groups due to the perceived threat of mass surveillance.^13^ According to the American Civil Liberties Union, approaches toward compulsory adoption of such applications are coercive and counterproductive.^14^ The voluntary installation of any such application has become almost a fundamental cornerstone for pronouncing judgment on their ethical validity.^15^ ^,^^16^ Any contact tracing mobile app that requires compulsory installation and collects user-data has been deemed incompatible with civil liberties and human rights, almost axiomatically.

Consequently, in nearly all major democracies, the installation of contact-tracing applications has been left to the discretion of the individual. However, in India, the largest democracy of the world, the installation of such an application (app), the Aarogya Setu (from Sanskrit “the bridge to health”)^17^ has been deemed mandatory in certain situations involving certain public interactions like attending government and private offices, visiting malls, and during movement within containment zones.^18^ ^,^^19^ E-commerce companies delivering products, food delivery aggregators, and some private outpatient health facilities have also made it mandatory for their employees or customers to install the app.^20^ However, some provisions like the installation of the app to avail domestic air travel were withdrawn on providing a self-declaration of good health, and the rule for compulsory installation for office employees was relaxed after criticism and legal challenges in courts of law.^21^

Previous ethical commentaries on the subject have focused on the ethical implications involved in developing and deploying such applications, but mostly in the context of the developed world.^22^ Furthermore, there are no case studies to assess the ethical impact in the event of mandatory deployment of such an application in a developing country threatened by the COVID-19 pandemic. In this commentary, we make the argument that the ethics of mandatory installation of a digital contact tracing app at the time of a pandemic needs to be viewed as a public health intervention at the time of a public health emergency. Furthermore, the ethical justification of such an intervention should be evaluated through an ethical framework similar to those that have warranted overriding individual autonomy in landmark public health interventions enabling health promotion and harm reduction for both the individual and their communities.^23^ ^,^^24^ ^,^^25^ We also argue that the demonstration of trust through an emphasis on transparency, predefined limits to data collection, usage, and data destruction timelines related to a contact tracing app should be adequate for instilling confidence in the reasonable individual even in the absence of voluntariness. Here, we confine ourselves to a case study of the Aarogya Setu application in India and discuss the ethical considerations involved in its mandatory adoption by mobile phone users.

## The Aarogya Setu Application: Assessing Privacy and Concerns

The Aarogya Setu App is a government of India backed mobile application which has already become the most downloaded healthcare app in the world.^26^ It has features for contact tracing, self-assessment of the user’s present health status, and informs those at-risk through notifications of the appropriate actions to be taken.^27^ Furthermore, the data transferred by the app enhance the preparedness of public health departments to mitigate the spread of the pandemic. The app, when installed on any smartphone, interacts through the Bluetooth interface of the device with that of another smartphone in its vicinity (the Bluetooth signal range), whereby encrypted tokens are exchanged.

The app also collects GPS location data of the phone at periodic intervals. The encrypted tokens and the GPS information are stored locally until the user tests positive for COVID-19.^28^ Although the positive report can be communicated to the server through self-reporting, it is not dependent on the user’s action since the Aarogya Setu server receives real-time backend data about positive COVID-19 cases from the Indian Council of Medical Research database. When the server receives an alert about a COVID-19 positive case, it queries the corresponding user’s Aarogya Setu application to fetch the locally stored encrypted tokens and GPS information to identify their contacts at potential risk and to the authorized public health personnel involved in contact tracing. However, the identity of the COVID-19 positive user is never revealed to any of their contacts.^29^

Questions have been raised on an apparent lack of clarity about the functioning of the app as well as the purported over-collection of user data, since the app collects both Bluetooth and GPS data, as opposed to only Bluetooth data in certain other apps like those based on the Google–Apple framework.^30^ Such technical issues can underpin key ethical concerns related to privacy infringement, data misuse, and anonymity, and their assessment is necessary to determine the ethical justifiability of such applications.^31^

In the case of the Aarogya Setu app, GPS data collection is necessary for hotspot identification and intimation to the user that they have passed through such an area by examining their travel history. If a user tests positive for COVID-19, the location data is used to map the places the user visited in the last 30 days to identify areas that need more elaborate testing and containment. Under regular use, the location data are stored as de-identified data and are correlated with the identifiable information only where a more detailed contact tracing of a COVID-19 positive user is required.^32^

Some people may not feel comfortable with the idea that the government agencies would be able to trace the complete location information of an infected patient over 30 days. Nevertheless, such a view is mostly redundant since, in any case, the very fact of using a mobile phone causes the automatic recording of one’s travel history (albeit with less location accuracy than GPS) via mobile phone towers of the mobile phone service providers. Hypothetically, if the government wanted to go rogue, its agencies could fairly easily get access to this data and correlate that with a user’s phone number to track them.

The storage of the data on a centralized government server has caused some to raise concerns about the security of the stored data, data access, and retention.^33^ Although data stored on a centralized government server lie exclusively under the government’s domain, nevertheless, it can offer greater security per se, compared to data stored on a public server, which could be open to various sorts of tampering and attacks. The counter-argument is such data should never be collected or stored without the individual’s consent, but that may not hold during a public health emergency when risk mitigation becomes paramount.

Nevertheless, as far as the data access rule and privacy are concerned, the Government of India has delineated what is stored and in what form, and who has access to it. Besides, the data retention periods are also laid out very explicitly in the Aarogya Setu privacy policy. For instance, server data for all users who have not tested positive for COVID-19 will be purged 45 days after they were uploaded, and the data for those who have tested positive will be removed 60 days after recovery.^34^ Although some may have an inherent distrust for government guarantees,^35^ the situation here is no different than any third-party (private) app, some of them run by powerful global corporations, where one has to trust the creators to adhere to the written privacy policy. The argument of voluntariness is also perhaps misplaced, since smartphone operating system software and some preinstalled applications by Google and Apple run by default on their supported smartphones, and disabling them would lead to a crippling loss of functionality of the device itself.

## The Justification of Public Health Interventions During a Pandemic

A pandemic constitutes a public health emergency where the achievement of public health goals for securing the health of the population faces severe, if not unprecedented, challenges. It is also understood that the “public” in “public health” is considered to represent the collective goals of societal health achievable through the application of government agency, which includes the applicability of the coercive powers of the state.^36^ Consequently, any delay in the implementation of a readily available and effective contact tracing app that would cause enormous avoidable health and economic costs may also be considered violating the principle of human rights.

Conflicts between moral considerations invariably arise when searching for ethical justification of public health actions. In this case, the relevant considerations include achieving maximum utility through public participation while balancing the need for respecting individual liberty, privacy, and confidentiality. In such moral conflicts, five justificatory conditions have been proposed by Childress et al.^37^ to help determine if public health promotion that overrides certain ethical considerations such as individual liberty is warranted. These conditions include effectiveness, proportionality, necessity, least infringement, and public justification. Similarly, Upshur^38^ also recommended four principles to assess when a public health action is justified: harm, least restrictive or coercive means, reciprocity, and transparency. Technology-assisted contact tracing can also be assessed through a similar ethical framework that incorporates these principles and considerations tempered with the immediacy of response required for controlling a pandemic.

## Beneficence and Effectiveness

Contact tracing applications are designed to benefit both the user through notifications alerting them of having come in contact with an infected person and the society by enabling early identification of disease clusters and formulation of effective public health measures to prevent further disease transmission.^39^ Although the effectiveness of contact tracing applications during pandemics does not yet have a robust body of supportive evidence, this paucity is expected, considering this is the first pandemic in an era of ubiquitous digital technology exemplified by the global penetration of smartphones and mobile internet. Nevertheless, according to the Government of India, based on preliminary data, it was found that among the Aarogya Setu app users who were recommended testing, 24% were found COVID-19 positive, nearly fivefold higher compared to the overall COVID-19 positivity rate in the country.^40^ The app is also helping civic authorities of the country to identify the disease trends and map containment zones within their cities.^41^

Consequently, the large-scale use of the application by the country’s mobile phone users is likely to be highly cost-effective if it can reasonably complement and mitigate the work involved in the labor-intensive manual tracing operations. Furthermore, for countries under lockdown, such apps could be utilized as a part of their exit or unlock strategies.^42^ The potential health-related and economic cost savings on extensive use of such an app would also meet the ethical principle of justice.

The necessity of the Aarogya Setu app is also reasonable and justified considering the persistently escalating case burden in the country.^43^ Moreover, its mandatory installation is a consequence of the requirement of uptake by a critical mass, possibly as high as 70% of all mobile phone users, to render it an overwhelmingly successful public health intervention for controlling the COVID-19 pandemic compared to standard interventions, further necessitating the need for a single contact tracing app.^44^ ^,^^45^

The alerts sent to app users who have been in contact with a confirmed case can also help them to go into early isolation and limit the spread of the infection to their susceptible household, neighborhood and work contacts, especially when the former are asymptomatic, a morally appealing scenario. The use of such an application is also foregrounded in reciprocity due to the reduced risk of harm to a potential user’s contacts that is not possible in the app’s absence as discussed previously.

## Demonstrating Trust and Alleviating Privacy Concerns, But to What Extent?

Legitimate concerns exist over surveillance through apps, especially in nondemocratic societies lacking civil rights for their citizens, where political dissidents may be tracked and suppressed using such apps.^46^ However, in functional democratic societies, with sufficient legal and constitutional safeguards, these concerns may be exaggerated since governments can be held accountable by an independent judiciary. Moreover, democratic governments are obliged to take measures to convince the people to trust that the government is acting in their best interests^47^ and foster their cooperation for adherence to such public health recommendations.^48^ For instance, in India, after the initial criticism, the government was responsive in rendering the Aarogya Setu app open-source.^49^ Together with the decision to announce a bounty program for ethical hackers to find any inadvertent security loopholes or privacy issues, it demonstrated a commitment to transparency.^50^

A pandemic situation does create conflicts over maintaining the highest possible privacy and the greater public good. Moreover, even in the absence of an app, infected patients are morally expected to truthfully reveal their exact travel history during a manual contact tracing interview to enable timely intimation to those who had come in contact with them and are at risk.

Furthermore, even in the unlikely instance of a data breach, as discussed previously, the worst-case scenarios need to be defined. For instance, leakage of the names and addresses of a COVID-19 patient may, unfortunately, result in the short-term stigmatization of the individual. However, although disconcerting, the level of stigma, in this case, is not comparable to that in chronic infective conditions like HIV-AIDS that are not associated with recovery.^51^

It is true that for some individuals, concerns for digital privacy may trump all other considerations,^52^ including those related to health, and mandatory installation of a contact tracing application may cause them acute psychological agony. Nevertheless, we argue that the responsibility of the government during a public health emergency is limited to satisfying the reasonable individual, a principle consistent with Mill’s classic harm principle where the “physical or moral good” of the individual is deemed able to be superseded if necessary for preventing “harm to others.”^53^ Moreover, just like any other justifiable public health intervention, such as making people wear helmets and seat-belts to reduce the risks to their lives, the government need not go overboard in convincing the last skeptic to change their opinion. This is particularly true for an intervention that does not upset the way of life of the people by imposing any significant restrictions.

## Overriding Liberty: Arguments from Analogy, Lack of Engagement, and Equity

It is well-established that promoting public health may legitimately override the concern of individual liberty in specific cases, even in countries with a strong ingrained culture of libertarian ethos and anti-paternalistic attitudes. Some examples include the ban on smoking in public places, mandatory helmets, and car seat-belt laws that have been successfully advocated, legislated, and implemented.^54^ ^,^^55^ Even mandatory vaccination of infants and children through coercive policies is likely to be ethically justifiable, notwithstanding the miniscule risk of vaccine-related injury.^56^ Moreover, when traveling to foreign lands, travelers sometimes must take compulsory vaccination for diseases like yellow fever as per international law, which can cause pronounced allergies and adverse effects in rare cases. One could argue that international travel is voluntary, but under certain circumstances like when visiting an ailing relative, it can be justified. Nevertheless, these analogies are limited by the absence of long-term harm potential compared to a hypothetical malicious contact tracing app that collects confidential information it was not supposed to collect, and uses it for purposes detrimental to the user.

Another valid concern with the compulsory installation of an application is the potential lack of engagement by the privacy-concerned users and accentuation of their vulnerability. Without trust in the health system, there can be an apprehension that suspicious users may apply means to subvert the functioning of these applications, like by turning off location services.^57^ However, in an age of smartphone addiction and nomophobia,^58^ the possibility of people leaving their homes without their primary phones and disconnecting location services may be more theoretical than practical arguments.

A more problematic challenge is the lack of smartphone accessibility among three out of every four Indians, with those not possessing a smartphone likely to be deprived of the benefits of the Aarogya Setu app.^59^ To achieve equity and justice, efforts toward the development and deployment of a nonsmartphone version of the app are therefore urgently warranted. Nevertheless, there exists a significant rural–urban disparity with accessibility to smartphones in India, so the use of the app in halting the spread of COVID-19 would be beneficial for at least the cities which have been hitherto comparatively worse affected than much of rural India.

## Conclusion

Installing a government-backed application for contact tracing during a pandemic is a legitimate public health intervention. However, such an intervention, although not fundamentally different from compulsorily wearing a helmet, seat belt, or taking a vaccine before travel, lacks precedent, since the potentially detrimental implications for the users can manifest even after much time has elapsed. Consequently, governments, especially in democratic societies, on their part should walk the extra mile to make reasonable efforts to instill confidence among their citizens for using these apps. Civil society organizations, although having the right to forewarn against data misuse by such apps, also have an added responsibility during pandemics of avoiding being adversely judgmental based on a reflexive distrust of the government. Nevertheless, ultimately, it will be the generation of first-class evidence proving the effectiveness of these apps in stopping the chain of transmission that can justify their mandatory use by the yardstick of public health ethics.

## Notes

1.

World Health Organization. Modes of transmission of virus causing COVID-19: implications for IPC precaution recommendations. *Scientific Brief*
; 2020 March 29; available at https://www.who.int/news-room/commentaries/detail/modes-of-transmission-of-virus-causing-covid-19-implications-for-ipc-precaution-recommendations (last accessed 26 Jun 2020).

2.

World Health Organization. Global surveillance for COVID-19 caused by human infection with COVID-19 virus; available at https://www.who.int/publications/i/item/global-surveillance-for-covid-19-caused-by-human-infection-with-covid-19-virus-interim-guidance (last accessed 26 Jun 2020).

3.

Greiner AL , Angelo KM , McCollum AM , Mirkovic K , Arthur R , Angulo FJ . Addressing contact tracing challenges-critical to halting Ebola virus disease transmission. International Journal of Infectious Diseases 2015;41:53–5.2654680810.1016/j.ijid.2015.10.025

4.

Gowda G , Holla R , Ramraj B , Gudegowda KS . Contact tracing and quarantine for COVID-19: Challenges in community surveillance. Indian Journal of Community Health 2020;32(2):306–8.

5.

Deccan Herald. Corona warriors attacked in Belagavi village; 2020 June 12; available at https://www.deccanherald.com/state/karnataka-districts/corona-warriors-attacked-in-belagavi-village-848840.html (last accessed 26 Jun 2020).

6.

World Health Organization. Transmission of COVID-19 by asymptomatic cases; 2020 June 11; available at http://www.emro.who.int/health-topics/corona-virus/transmission-of-covid-19-by-asymptomatic-cases.html (last accessed 26 Jun 2020).

7.

Hart V , Siddarth D , Cantrell B , Tretikov L , Eckersley P , Langford J , et al. 2020 *Outpacing the Virus: Digital Response to Containing the Spread of COVID-19 while Mitigating Privacy Risks*; available at https://ethics.harvard.edu/outpacing-virus (last accessed 26 June 2020).

8.

Ferretti L , Wymant C , Kendall M , Zhao L , Nurtay A , Abeler-Dörner L , et al. 2020 Quantifying SARS-CoV-2 transmission suggests epidemic control with digital contact tracing. Science 368(6491):eabb6936.3223480510.1126/science.abb6936PMC7164555

9.

Garg S , Bhatnagar N , Gangadharan N . A case for participatory disease surveillance of the COVID-19 pandemic in India. JMIR Public Health Surveillance 2020;6(2):e18795.3228703810.2196/18795PMC7164788

10.

MIT Technology Review: COVID Tracing Tracker; 2020 June 15; available at https://www.technologyreview.com/2020/05/07/1000961/launching-mittr-covid-tracing-tracker/ (last accessed 26 Jun 2020).

11. See note 10, MIT Technology Review (2020).

12.

Press Information Bureau. *Aarogya Setu is Now Open Source*; 2020 May 26; available at https://pib.gov.in/PressReleseDetailm.aspx?PRID=1626979# (last accessed 26 Jun 2020).

13.

Ranisch R , Nijsingh N , Ballantyne A , Buyx A , Friedrich O , Hendl T , et al. Ethics of digital contact tracing apps for the Covid-19 pandemic response. *Kompetenznetz Public Health COVID-19*; 2020; available at https://www.public-healthcovid19.de/images/2020/Ergebnisse/Ethics_of_digital_contact_tracing-v1.pdf (last accessed 26 Jun 2020).

14.

Guliani NS . *ACLU White Paper—Government Safeguards for Tech-Assisted Contact Tracing*; available at https://www.aclu.org/other/aclu-white-paper-government-safeguards-tech-assisted-contact-tracing (last accessed 26 Jun 2020).

15.

O’Neill HP . *India is Forcing People to Use its Covid App, Unlike any Other Democracy*; 2020 May 7; available at https://www.technologyreview.com/2020/05/07/1001360/india-aarogya-setu-covid-app-mandatory (last accessed 26 Jun 2020).

16.

Morley J , Cowls J , Taddeo M , Floridi L . Ethical guidelines for COVID-19 tracing apps. Nature 2020;582(7810):29–31.3246759610.1038/d41586-020-01578-0

17. Aarogya Setu; available at https://www.mygov.in/aarogya-setu-app/ (last accessed 26 Jun 2020).

18.

Indian Express. *Aarogya Setu Mandatory: Who All Must Download the App Right Away 2020*; 2020 May 3; available at https://indianexpress.com/article/technology/social/aarogya-setu-app-mandatory-contact-tracing-app-6389284 (last accessed 26 Jun 2020).

19. See note 15, O’Neill 2020.

20.

Shashidhar KJ . *Aarogya Setu App and Its Many Conflicts*; 2020; available at https://www.orfonline.org/expert-speak/aarogya-setu-app-many-conflicts-67442/ (last accessed 26 Jun 2020).

21.

Deccan Chronicle. *Aarogya Setu App not Mandatory but Show COVID-19 Negative Certificate Itself*; 2020 May 24; available at https://www.deccanchronicle.com/nation/current-affairs/240520/hardeep-puri-aarogya-setu-app-not-mandatory-self-declaration-form-al.html (last accessed 26 Jun 2020).

22.

Parker MJ , Fraser C , Abeler-Dörner L , Bonsall D . Ethics of instantaneous contact tracing using mobile phone apps in the control of the COVID-19 pandemic. Journal of Medical Ethics 2020;46(7):427–31.3236670510.1136/medethics-2020-106314PMC7231546

23.

Oriola TA . Ethical and legal analyses of policy prohibiting tobacco smoking in enclosed public spaces. Journal of Law and Medical Ethics 2009;37(4):828–40.10.1111/j.1748-720X.2009.00454.x20122119

24.

Jones MM , Bayer R . Paternalism and its discontents: Motorcycle helmet laws, libertarian values, and public health. American Journal of Public Health 2007;97(2):208–17.1719485610.2105/AJPH.2005.083204PMC1781413

25.

Giubilini A , Savulescu J . Vaccination, risks, and freedom: The seat belt analogy. Public Health Ethics 2019;12(3):237–49.3208241810.1093/phe/phz014PMC7020768

26.

Press Trust of India. *Aarogya Setu Most Downloaded Healthcare App in World: Amitabh Kant*; 2020 May 8; available at https://economictimes.indiatimes.com/tech/internet/aarogya-setu-most-downloaded-healthcare-app-in-world-amitabh-kant/articleshow/75633564.cms?from=mdr (last accessed 26 Jun 2020).

27.

Government of India. *Aarogya Setu Mobile App. Aarogya Setu FAQs*; 2020; available at https://static.mygov.in/rest/s3fs-public/mygov_159056978751307401.pdf (last accessed 26 Jun 2020).

28.

Government of India. *Aarogya Setu Mobile App. Aarogya Setu Technical FAQs*; 2020; available at https://static.mygov.in/rest/s3fs-public/mygov_159056968451307401.pdf (last accessed 26 Jun 2020).

29.

Times of India. *Aarogya Setu App Does not Access Your Data Unless You have COVID-19, Says NITI Aayog's Arnab Kumar*; 2020 May 10; available at https://timesofindia.indiatimes.com/home/sunday-times/all-that-matters/aarogya-setu-app-does-not-access-your-data-unless-you-have-covid-19-says-niti-aayogs-arnab-kumar/articleshow/75650510.cms (last accessed 26 June 2020).

30.

George PJ . Coronavirus | What are the concerns around the Aarogya Setu app? *The Hindu*
; 2020 April 26; available at https://www.thehindu.com/sci-tech/technology/coronavirus-what-are-the-concerns-around-the-aarogyasetu-app/article31434768.ece (last accessed 26 June 2020).

31. See note 16, Morley et al. 2020, at 29–31.

32.

Government of India. *Aarogya Setu Privacy Policy*; 2020; available at https://static.mygov.in/rest/s3fs-public/mygov_159051645651307401.pdf (last accessed 26 June 2020).

33. See note 20, Shashidhar 2020.

34. See note 32, Government of India 2020.

35.

Xafis V , Schaefer GO , Labude MK , Zhu Y , Hsu LY . The perfect moral storm: Diverse ethical considerations in the COVID-19 pandemic. Asian Bioethics Review 2020;12:65–83.10.1007/s41649-020-00125-3PMC725563532837550

36.

Childress JF , Faden RR , Gaare RD , Gostin LO , Kahn J , Bonnie RJ , et al. Public health ethics: mapping the terrain. The Journal of Law, Medicine & Ethics 2002;30(2):170–8.10.1111/j.1748-720x.2002.tb00384.x12066595

37. See note 36, Childress et al. 2002, at 170–8.

38.

Upshur RE . Principles for the justification of public health intervention. Canadian Journal of Public Health 2002;93(2):101–3.1196817910.1007/BF03404547PMC6979585

39. See note 13, Ranisch et al. 2020.

40. See note 12, Press Information Bureau 2020.

41.

Patel L. Aarogya Setu app data is helping AMC identify potential hotspots. *Ahmedabad Mirror*; 2020 June 15; available at https://ahmedabadmirror.indiatimes.com/ahmedabad/others/aarogya-setu-app-data-is-helping-amc-identify-potential-hotspots/articleshow/76376752.cms (last accessed 26 Jun 2020).

42.

Resnick B. Social distancing can’t last forever. Here’s what should come next. *Vox*; 2020 March 30; available at https://www.vox.com/science-and-health/2020/3/26/21192211/coronavirus-covid-19-social-distancingend (last accessed 26 Jun 2020).

43.

Government of India. *Ministry of Health and Family Welfare*; 2020; available at https://www.mohfw.gov.in/ (last accessed 26 Jun 2020).

44.

Bassi A , Arfin S , John O , Jha V . An overview of mobile applications (apps) to support the coronavirus disease 2019 response in India. Indian Journal of Medical Research 2020;151:468–73.3247455710.4103/ijmr.IJMR_1200_20PMC7530460

45. See note 7, Hart et al. 2020.

46. See note 35, Xafis et al. 2020, at 65–83.

47.

Hall MA , Dugan E , Zheng B , Mishra AK . 2001 Trust in physicians and medical institutions: What is it, can it be measured, and does it matter? Milbank Quarterly 79(4):613–39.1178911910.1111/1468-0009.00223PMC2751209

48.

van der Weerd W , Timmermans DR , Beaujean DJ , Oudhoff J , van Steenbergen JE . Monitoring the level of government trust, risk perception and intention of the general public to adopt protective measures during the influenza A (H1N1) pandemic in The Netherlands. BMC Public Health 2011;11:575.2177129610.1186/1471-2458-11-575PMC3152536

49. See note 12, Press Information Bureau 2020.

50.

Government of India. *Aarogya Setu Bug Bounty Program*; 2020; available at https://static.mygov.in/rest/s3fs-public/mygov_159057669351307401.pdf (last accessed 26 Jun 2020).

51.

Mahajan AP , Sayles JN , Patel VA , Remien RH , Sawires SR , Ortiz DJ , et al. Stigma in the HIV/AIDS epidemic: A review of the literature and recommendations for the way forward. AIDS 2008;2(Suppl 2):S67–79.10.1097/01.aids.0000327438.13291.62PMC283540218641472

52. See note 22, Parker et al. 2020, at 427–31.

53.

Holtug N. The harm principle. Ethical Theory and Moral Practice 2002:5;357–89.

54.

Eltorai AE , Simon C , Choi A , Hsia K , Born CT , Daniels AH . Federally mandating motorcycle helmets in the United States. BMC Public Health 2016;16:242.2695580610.1186/s12889-016-2914-3PMC4784405

55. See note 23, Oriola, at 828–40.

56. See note 25, Giubilini, Savulescu 2019, at 237–49.

57.

Granick JS . *Apple and Google Announced a Coronavirus Tracking System. How Worried Should We Be?* 2020 April 16; available at https://www.aclu.org/news/privacy-technology/apple-and-google-announced-a-coronavirus-tracking-system-how-worried-should-we-be/ (last accessed 26 Jun 2020).

58.

Sohn S , Rees P , Wildridge B , Kalk NJ , Carter B . Prevalence of problematic smartphone usage and associated mental health outcomes amongst children and young people: A systematic review, meta-analysis and GRADE of the evidence. BMC Psychiatry 2019;19(1):356.3177963710.1186/s12888-019-2350-xPMC6883663

59.

Nalapat S. Aarogya Setu: Are India’s non-smartphone users at risk of being left in the cold? *Timesnownews*; 2020 May 13; available at https://www.timesnownews.com/technology-science/article/aarogya-setu-are-india-s-non-smartphone-users-at-risk-of-being-left-in-the-cold/591350 (last accessed 26 Jun 2020).

